# Patients’ risk factors for periprosthetic joint infection in primary total hip arthroplasty: a meta-analysis of 40 studies

**DOI:** 10.1186/s12891-021-04647-1

**Published:** 2021-09-12

**Authors:** Xiaolei Ren, Lin Ling, Lin Qi, Zhongyue Liu, Wenchao Zhang, Zhimin Yang, Wanchun Wang, Chao Tu, Zhihong Li

**Affiliations:** Department of Orthopaedics, Hunan Key Laboratory of Tumor Models and Individualized Medicine, The Second Xiangya Hospital, Central South University, Changsha, Hunan P.R. China

**Keywords:** Periprosthetic joint infection, PJI, Total hip arthroplasty, THA, Risk factors

## Abstract

**Background:**

Periprosthetic joint infection (PJI) is a catastrophic complication after total hip arthroplasty (THA). Our meta-analysis aimed to identify the individual-related risk factors that predispose patients to PJI following primary THA.

**Methods:**

Comprehensive literature retrieval from Pubmed, Web of Science, and the Cochrane Library was performed from inception to Feb 20th, 2021. Patient-related risk factors were compared as per the modifiable factors (BMI, smoke and alcohol abuse), non-modifiable factors (gender, age), and medical history characteristics, such as diabetes mellitus (DM), avascular necrosis (AVN) of femoral head, femoral neck fracture, rheumatoid arthritis (RA), cardiovascular disease (CVD), and osteoarthritis (OA) etc. The meta-analysis was applied by using risk ratios with 95% corresponding intervals. Sensitivity analysis and publication bias were performed to further assess the credibility of the results.

**Results:**

Overall, 40 studies with 3,561,446 hips were enrolled in our study. By implementing cumulative meta-analysis, higher BMI was found associated with markedly increased PJI risk after primary THA [2.40 (2.01–2.85)]. Meanwhile, medical characteristics including DM [1.64 (1.25–2.21)], AVN [1.65 (1.07–2.56)], femoral neck fracture [1.75 (1.39–2.20)], RA [1.37 (1.23–1.54)], CVD [1.34 (1.03–1.74)], chronic pulmonary disease (CPD) [1.22 (1.08–1.37)], neurological disease [1.19 (1.05–1.35)], opioid use [1.53 (1.35–1.73)] and iron-deficiency anemia (IDA) [1.15 (1.13–1.17)] were also significantly correlated with higher rate of PJI. Conversely, dysplasia or dislocation [0.65 (0.45–0.93)], and OA [0.70 (0.62–0.79)] were protective factors. Of Note, female gender was protective for PJI only after longer follow-up. Besides, age, smoking, alcohol abuse, previous joint surgery, renal disease, hypertension, cancer, steroid use and liver disease were not closely related with PJI risk.

**Conclusion:**

Our finding suggested that the individual-related risk factors for PJI after primary THA included high BMI, DM, AVN, femoral neck fracture, RA, CVD, CPD, neurological disease, opioid use and IDA, while protective factors were female gender, dysplasia/ dislocation and OA.

**Supplementary Information:**

The online version contains supplementary material available at 10.1186/s12891-021-04647-1.

## Background

Total hip arthroplasty (THA) has served as a successful elective surgical procedure that provides pain relief, restores joint function, and consequently enhances overall quality-of-life for millions of patients worldwide [1–3]. Although most patients benefit significantly from this advanced technique, there is still a minority of patients may suffer with device failure and thereby need additional operations [4, 5].

Periprosthetic joint infection (PJI) is defined as infection involving the joint prosthesis and adjacent tissue. Its incidence is rare, which has been reported to range from 0.25 to 2.0% [6]. However, this morbidity may be devastating since it could jeopardize the results of the procedure, and even increase mortality [1, 7]. Currently, tremendous efforts have been implemented and improvements in prosthesis material and surgical technique have been achieved in THA [8]. However, the incidence of PJI is still increasing worldwide alongside the uprising prevalence of revision surgery, morbid obesity epidemic, and other comorbidities. Treatments for PJI usually comprise prolonged systemic antibiotic treatment, debridement and revision, which may pose substantial burden of compromised function and impaired quality of life to the patients [1, 6]. Moreover, patients with PJI are also associated with higher financial burden and greater medical resource utilization on the health care system [4, 9, 10]. Accordingly, identification of potential risk factors is of great importance for early detection and reduction of the deleterious consequence that attributed to PJI.

Nowadays, attention has been directed toward a plausible link between PJI and patients’ individual risk factors in addition to the surgical- and hospital-related factors. More recently, substantial studies regarding the patient-related risk factors for PJI, such as obesity [4, 11–14], diabetes mellitus (DM) [14–17], male gender [18], rheumatoid arthritis (RA) [19, 20], and pulmonary diseases [6, 21], have been reported. However, the power of these studies may be impaired due to limited numbers of recruited patients, controversial results and range of potential risk factor studies. Besides, many of these risk factors were updated and even revised in recent years, which may consolidate or oppose against the previous results. Moreover, optimization of modifiable risk factor for PJI should be emphasized in clinical practice, while only limited studies have separated the modifiable and non-modifiable risk factors to PJI. Given the limitations and remained discrepancies abovementioned, we took into account all the available literature and performed this updated meta-analysis, aiming to identify the potential individual risk factors for PJI undergoing primary THA.

## Materials and methods

### Literature search strategy

The MOOSE (Meta-analysis of Observational Studies in Epidemiology) and PRISMA (Preferred Reporting Items for Systematic reviews and meta-Analysis) 2020 Checklist were followed for the completion of this study [22, 23]. The study authors did not supply relevant information.

A computer-aided systematic literature retrieval was performed without restriction of language. Databases including Pubmed, Web of Science, and the Cochrane Library were searched from inception to Feb 20th, 2021, with search terms in variably combinations listed as follows: (“PJI” OR “periprosthetic joint infection” OR “prosthetic joint infection” or “deep infection” OR “deep surgical site infection”) AND (“surgical” OR “surgical approach” OR “surgical incision” OR “incision”) AND (“THA” OR “THR” OR “total hip arthroplasty” OR “total hip replacement”). Specially, only primary THAs were included in this meta-analysis.

Additionally, cross-references checking of each enrolled study were manually performed to identify possible additional articles.

Articles with potentially relevant titles and abstracts were screened and scored firstly by two independent investigators (CT and XLR). A third investigator (LQ) was consulted in the event of discrepancies between the two reviewers. Afterwards, the full manuscripts may be read if the studies could meet the inclusion criteria and the relevant information will be extracted.

### Study selection

Included studies met the following criteria: 1) Quantitative observational studies including prospective cohorts or retrospective case-control trials; 2) Studies reported odds ratio (ORs) or risk ratio (RRs) for dichotomous risk factors and mean difference for continuous factors, or allowed calculation of ORs/ RRs from sufficient raw data; and 3) Risk factors must be demographic, comorbid, behavioral, infectious, native joint disease, other patient-related risk factors.

Exclusion criteria were as follows: 1) Insufficient data to estimate a pooled RR; 2) Duplicated data from the same authors, excluding the earlier and small studies; 3) Not focused on risk factors of PJI; 4) Documents without original raw data, such as correspondences, editorial materials, and reviews; 5) Not related to THA; and 6) Superficial infection.

### Data extraction and methodological quality assessment

Two investigators carefully reviewed and screened the titles and abstracts independently to identify eligible studies as per the inclusion and exclusion criteria. The full text was read if necessary. Information items extracted from enrolled trials included as follows: first author, year of publication, location and investigation year of the study, study design, duration of follow-up (years), number of participants involved, confounders adjusted, age, and gender. Demographic risk factors, including behavioral risk factors, comorbid conditions, native joint disease, and other patient-related risk factors were searched for PJI.

Quality assessment was performed by using the Newcastle-Ottawa Scale (NOS) as previously described [24].

### Sensitivity analysis

Sensitivity analysis was used to assess the robustness of the results and was planned based on the risk of bias assessment. A sensitivity analysis was performed for all the risk factors with substantial heterogeneity. Meta-regression was done for gender and DM.

### Statistical analysis

This meta-analysis was conducted by using the STATA software (Stata 12.0) and Review Manager (RevMan 5.3). Due to variability in the populations and risk factors of included studies, random-effects models were adopted for meta-analysis. Data were categorized and analyzed by groups of risk factors.

The heterogeneity across the eligible studies was quantified using *I*^*2*^ statistic. Meta-regression was done to explore heterogeneity and determine the causes of heterogeneity.

## Results

### Search results and study identification

**Fig. 1:**
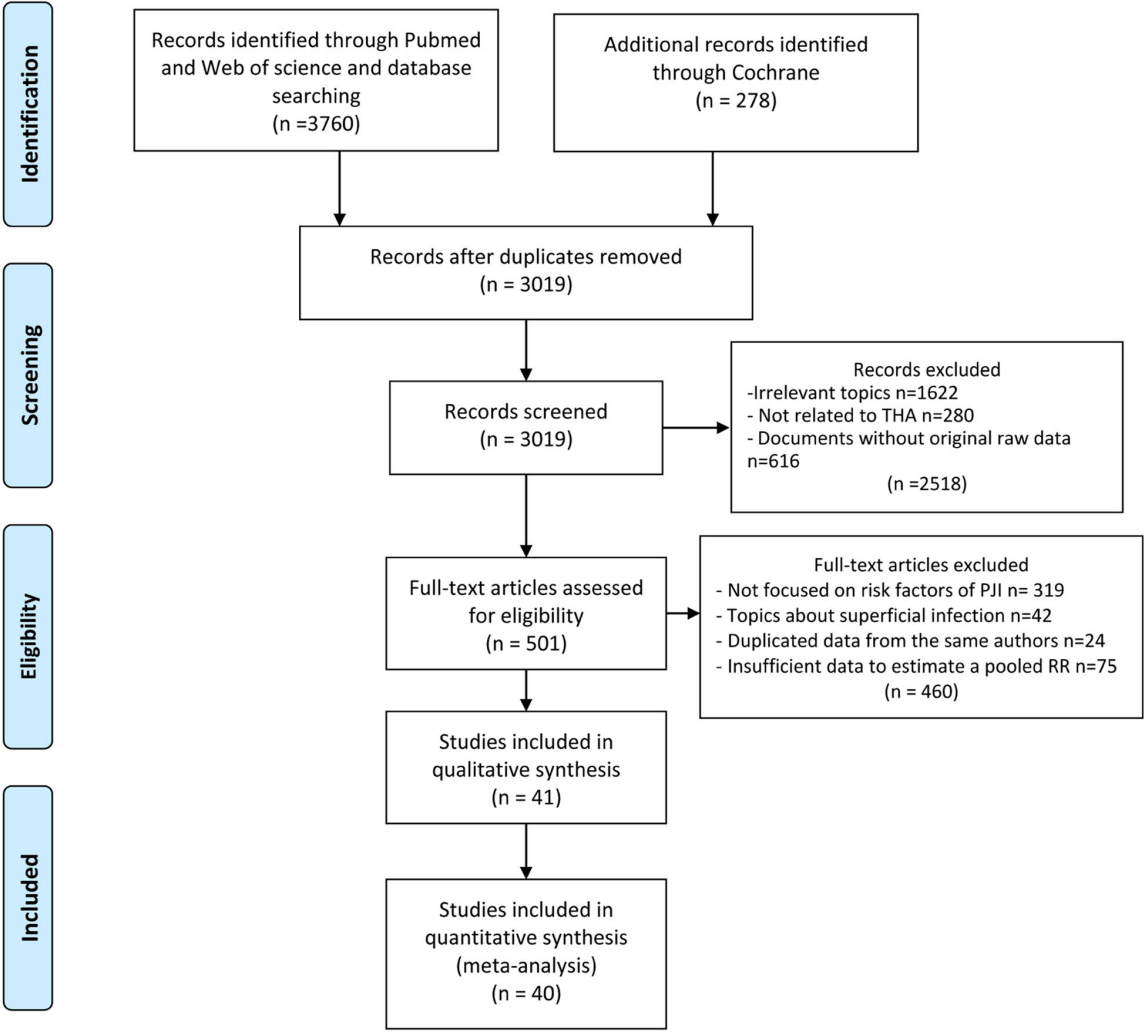
PRISMA Flow diagram showing the study selection process

A total of 4038 articles were collected by search strategy from PubMed, Web of science, and the Cochrane library. After assessment according to the inclusion eligibility criteria, 40 studies comprising 3,561,446 hips in total were finally recruited in this meta-analysis (Fig. 1). Articles with irrelevant topics like those without risk factors, reviews, meeting abstracts and papers with duplicate reports or unavailable data were excluded. Particularly, 75 studies which investigated risk factors of PJI both in total knee arthroplasty (TKA) and THA were excluded because the data of only primary THA could not be extracted. Among the included articles, eight were published in Asia-Pacific region, including Australia, Korea and Malaysia. Besides, seventeen studies were performed in the USA and sixteen in Europe including UK, Finland, Spain, Italy, Netherlands, Switzerland, Belgium and Portugal. All studies were published between 2001 and 2020 except two performed by Surin [25] and Vannini [15], which were published in 1983 and 1984, respectively. The investigation years ranged from 1969 to 2018. Study design included prospective case-control, prospective cohort, retrospective case-control and retrospective cohort studies. The mean ± standard deviation of follow-up time was 2.92 ± 2.62 years. The methodological quality was evaluated by NOS scores and calculated total quality scores ranged from 5 to 8 (Table S1). After qualitative synthesis, Yong et al. was excluded because of the low quality and low incidence of PJI, which might cause the bias [26]. All the characteristics details of included studies were demonstrated in Table 1.

**Table 1:** Demographic characteristics of the enrolled studies

ID	Authors (Year)	Location	Investigation years	Study design	Follow-up years	Sample size, n	Confounders adjusted	NOS
1	Bozic, et al. (2014) [27]	North America	1990–2011	Prospective case-control	3.7	587	Multivariate	7
2	Burn, et al. (2018) [19]	Europe	1995–2014	Retrospective cohort	0.25	10,260	Multivariate	7
3	Chee, et al. (2010) [28]	Europe	1998–2003	Prospective cohort	5	110	Univariate	5
4	Choong, et al. (2007) [29]	Asia-Pacific	1998–2004	Retrospective cohort	3.5	819	Univariate	5
5	Cordero-Ampuero, et al. (2010) [30]	Europe	1997–2007	Prospective case-control	2.3	124	Univariate	5
6	Dale, et al. (2011) [31]	Europe	2005–2009	Prospective cohort	1	5540	Multivariate	7
7	Davis, et al. (2011) [32]	Europe	1998–2005	Prospective cohort	5	1617	Multivariate	8
8	de Boer, et al. (2001) [33]	Europe	1996–1999	Retrospective cohort	1	12,588	Univariate	6
9	Dowsey, et al. (2008) [12]	Asia-Pacific	1998–2005	Retrospective cohort	1	1207	Univariate	5
10	Gittings, et al. (2017) [34]	North America	2009–2014	Retrospective cohort	1	33	Univariate/ multivariate	6
11	Gonzalez, et al. (2018) [35]	Europe	1996–2013	Prospective cohort	5.6	5198	Multivariate	7
12	Hartford, et al. (2020) [36]	North America	–	Retrospective cohort	1	1808	Multivariate	6
13	Huotari, et al. (2007) [37]	Europe	2001–2004	Retrospective cohort	1	5614	Univariate	5
14	Jamsen, et al. (2012) [14]	Europe	2002–2008	Retrospective cohort	1	3266	Multivariate	7
15	Jung, et al. (2017) [38]	Asia-Pacific	2013–2015	Retrospective cohort	0.25	10,690	Multivariate	6
16	Kildow, et al. (2017) [16]	North America	2005–2012	Retrospective case-control	2	61,778	Multivariate	6
17	Kurtz, et al. (2018) [39]	North America	2005–2015	Retrospective case-control	4.7	1,158,742	Multivariate	7
18	Lenguerrand, et al. (2018) [40]	Europe	2003–2013	Prospective cohort	4.6	623,253	Multivariate	8
19	Lubbeke, et al. (2007) [41]	Europe	1996–2005	Prospective cohort	5	2495	Multivariate	7
20	Maoz, et al. (2015) [42]	North America	2009–2011	Retrospective cohort	2	3672	Multivariate	6
21	Marjo, et al. (2007) [43]	Europe	2000–2002	Prospective cohort	1	1922	Multivariate	7
22	Matar, et al. (2020) [44]	North America	2012–2018	Retrospective cohort	1	7270	Multivariate	7
23	McIntosh, et al. (2006) [45]	North America	1998–2002	Retrospective cohort	2.7	448	Multivariate	6
24	Meermans, et al. (2012) [46]	Europe	1998–2006	Retrospective cohort	5.9	364	Univariate	5
25	Muilwijk, et al. (2006) [47]	Europe	1996–2003	Retrospective cohort	1	26,127	Multivariate	7
26	Namba, et al. (2012) [48]	North America	2001–2009	Retrospective cohort	1	30,491	Univariate/ multivariate	7
27	Ong, et al. (2009) [49]	North America	1997–2006	Prospective cohort	10	39,929	Multivariate	7
28	Peel, et al. (2011) [50]	Asia-Pacific	2000–2007	Prospective case-control	1	108	Univariate	5
29	Rondon, et al. (2018) [51]	North America	2000–2016	Retrospective case-control	5.3	145	Univariate	6
30	Sequeira, et al. (2020) [52]	North America	2005–2014	Retrospective cohort	1	741,078	Multivariate	6
31	Sequeira,S et al. (2020) [53]	North America	2005–2014	Retrospective cohort	1	483,405	Multivariate	6
32	Smith, et al. (2018) [54]	Asia-Pacific	2000–2014	Prospective cohort	1	91,585	Multivariate	7
33	Sodhi,N et al. (2020) [55]	North America	2005–2014	Retrospective cohort	2	14,944	Univariate	6
34	Sodhi, et al. (2020) [56]	North America	2005–2014	Retrospective cohort	2	162,489	Multivariate	6
35	Song, et al. (2012) [57]	Asia-Pacific	2006–2009	Retrospective cohort	1	3422	Univariate/ multivariate	7
36	Surin, et al. (1983) [25]	Europe	1970–1977	Retrospective cohort	3–10	803	Univariate	5
37	Tai, et al. (2014) [58]	Asia-Pacific	1997–2003	Prospective cohort	10	1420	Univariate	5
38	Triantafyllopoulos, et al. (2018) [59]	North America	1999–2013	Retrospective cohort	8.6	36,494	Univariate/ multivariate	7
39	Vannini, et al. (1984) [15]	Europe	1969–1979	Prospective case-control	2.9	1042	Univariate	5
40	Wilson, et al. (2020) [60]	North America	2009–2017	Retrospective cohort	1	8559	Multivariate	6

### Effect of modifiable individual factors on the incidence of PJI

**Fig. 2:**
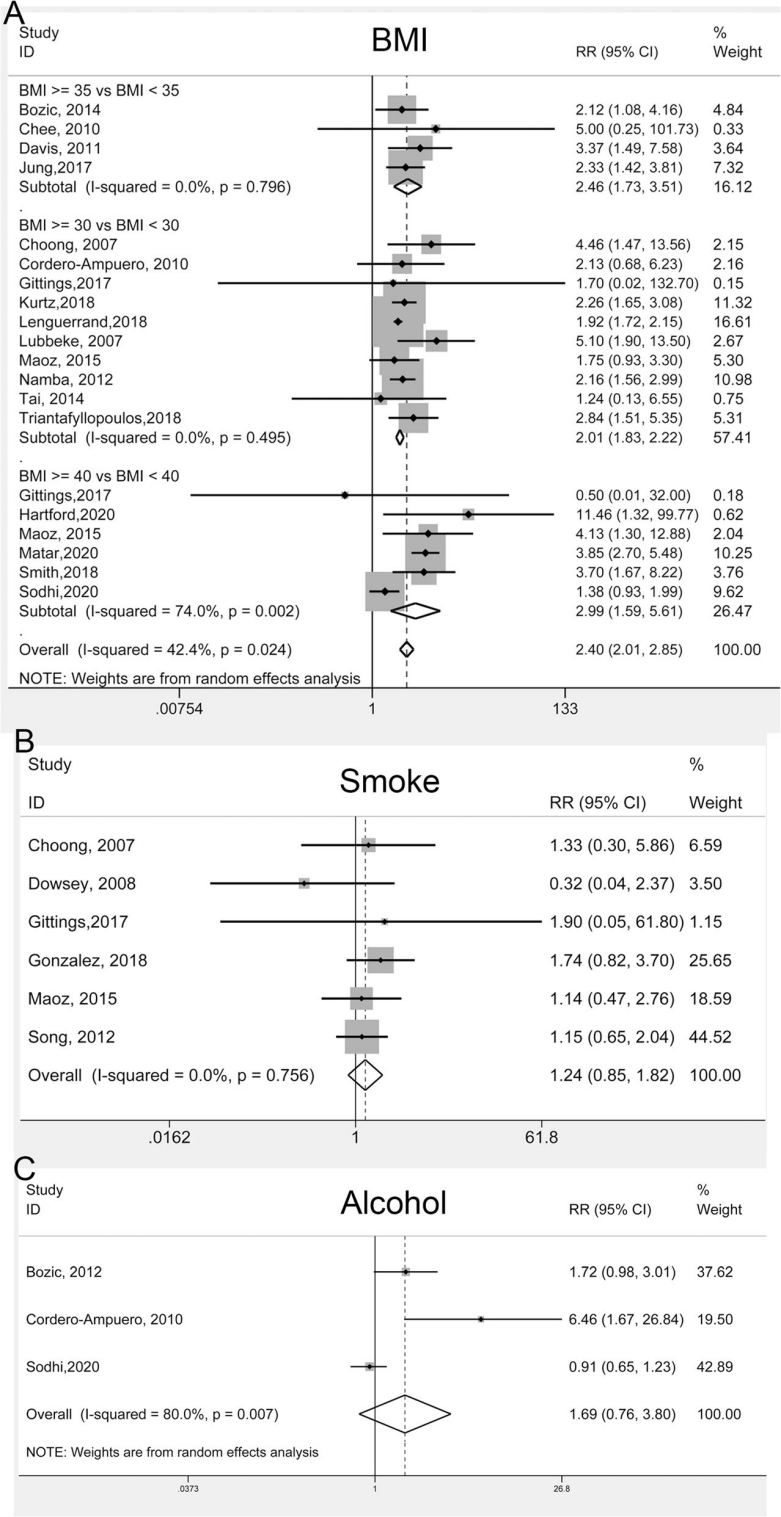
Forest plots of the meta-analysis of modifiable risk factors, including BMI (**A**), smoking (**B**), and alcohol abuse (**C**) as risk factors for periprosthetic joint infection (PJI) following total hip arthroplasty (THA). The diamond squares represent the pooled risk ratios (RRs) and corresponding 95% confidence intervals (CIs), while the squares and horizontal lines demonstrate the proportional weight and 95% CI of each included study, respectively

Modifiable individual risk factors among studies were compared in terms of BMI (body mass index), smoke and alcohol abuse. Twenty-one studies were included in the BMI comparison. Subgroup analyses were further adopted according to different cut-off values, in which the patients were stratified by 30, 35 and 40 kg/m^2^ cut-off value group. All the pooled RRs and 95% CI were shown in Table 2. In all the subgroups based on BMI cut-off value, the results indicated that higher BMI was associated with higher incidence of PJI (Fig. 2A). The pooled RR (95% CI) of smoke and alcohol abuse (> 45 g/day males,> 30 g/day females) comparisons were 1.24 (0.85–1.82) (Fig. 2B) and 1.69 (0.76–3.80) (Fig. 2C), respectively, which suggested that smoke and alcohol abuse were not risk factors relating to PJI for THA.

**Table 2:** Modifiable risk factors for periprosthetic joint infection and the outcomes of meta-analysis

Comparisons	Number of studies	RR (95% CI)	Heterogeneity ***I***^**2**^, ***p***-value*
1. BMI (kg/m^2^)	20	2.40 (2.01–2.85)	42.4%, 0.024
BMI ≥30 vs. BMI < 30	10	2.01 (1.83–2.22)	0.0%, 0.495
BMI ≥35 vs. BMI < 35	4	2.46 (1.73–3.51)	0.0%, 0.796
BMI ≥40 vs. BMI < 40	6	2.99 (1.59–5.61)	74.0%, 0.002
2. Smoke vs. non	6	1.24 (0.85–1.82)	0.0%, 0.756
3. Alcohol abuse vs. non	3	1.69 (0.76–3.80)	80.0%, 0.007

The heterogeneity in BMI and smoke analysis groups were not significant (*I*^*2*^ < 50%). Although alcohol comparisons results exhibited significant heterogeneity (*I*^*2*^ = 80.0%), the few numbers of the included studies were not enough to analyze the source of heterogeneity.

### Effect of non-modifiable individual factors on the incidence of PJI

**Fig. 3:**
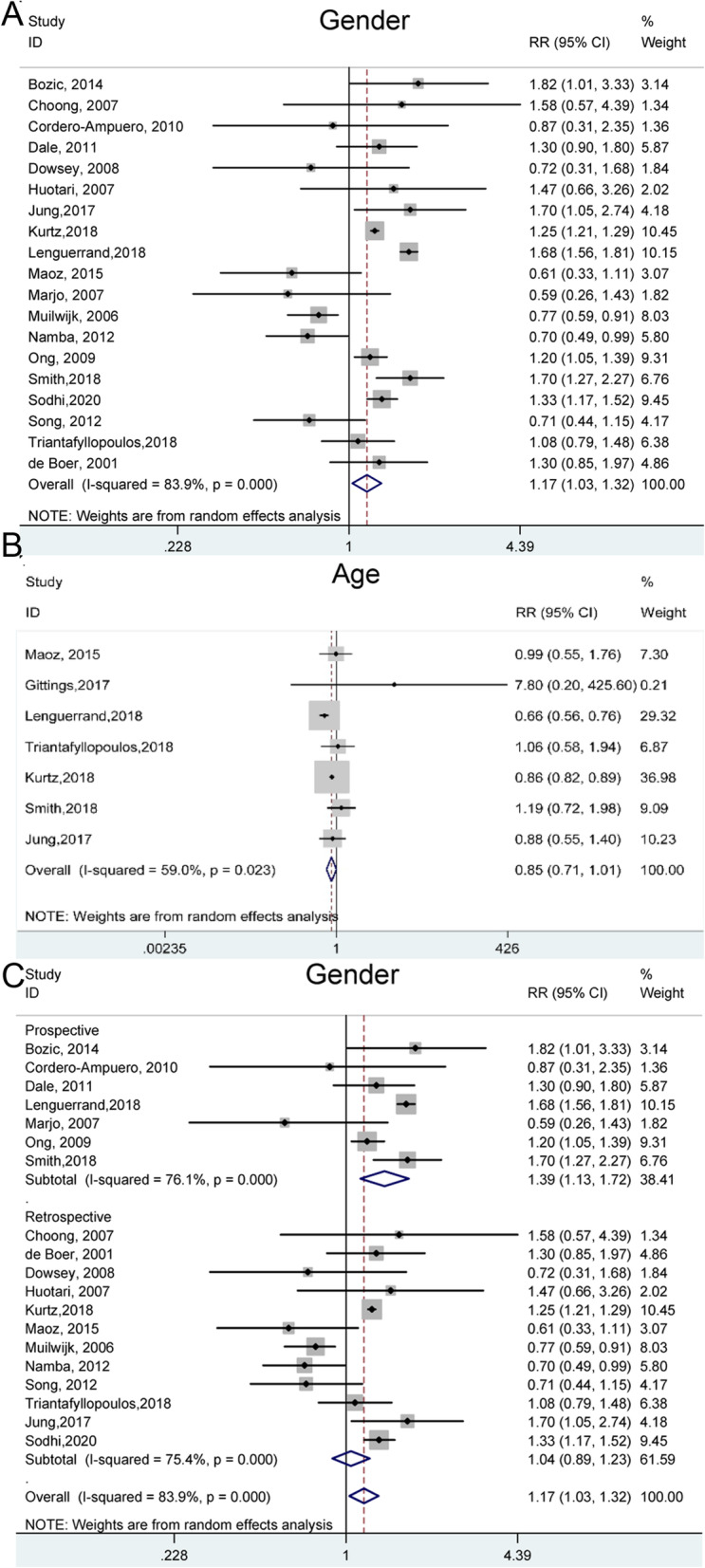
Forest plots of the meta-analysis of non-modifiable risk factors, including gender (**A**) and age (**B**) as risk factors for PJI following THA. Subgroup analysis for gender with different study design (**C**) were further shown to identify the possible association between gender with PJI

Gender and age were analyzed as non-modifiable individual factors to explore their effects on the incidence of PJI. The meta-analysis of gender factor included data from 19 studies. The forest plot of overall pooled RRs (Fig. 3A) showed significant difference in the prevalence of PJI between the male and female groups (RR = 1.17, 95% CI: 1.03–1.32) under a random-effects model, which suggested that female maybe a protective role of PJI for THA. Since obvious heterogeneity was observed (*I*^*2*^ = 83.9%, *p* < 0.001), subgroup analyses were further performed to identify the possible varieties, in which the patients were stratified by location, follow-up duration (years), study design and confounders adjustment (Table 3). Univariate meta-regressions showed significant relevance between the heterogeneity of gender factor and study design (*p* = 0.078). Seven studies from prospective studies group revealed that male had higher risk suffering from PJI than female (1.39, 1.13–1.72). Whereas, retrospective study group still had no significant difference (1.04, 0.89–1.23) (Fig. 3C). Besides, female gender also was a protective factor (1.16, 1.02–1.32) of PJI after THA in North American people, as shown in Figure S1A.

**Table 3:** Non- modifiable risk factors for periprosthetic joint infection and the outcomes of meta-analysis

Comparisons	Number of studies	RR (95% CI)	Heterogeneity ***I***^**2**^, ***p***-value*	***p***-value^**#**^
1. Gender	19	1.17 (1.03–1.32)	83.9%, < 0.001	
***1.1 Location***				0.804
North America	7	1.16 (1.02–1.32)	68.8%, 0.004	
Asia-Pacific	5	1.22 (0.80–1.87)	67.7%, 0.015	
Europe	7	1.13 (0.78–1.63)	88.5%, < 0.001	
***1.2 Follow-up years***				0.787
≥ 3	6	1.35 (1.13–1.62)	91.0%, < 0.001	
< 3	13	1.16 (1.00–1.33)	56.6%, 0.006	
***1.3 Study design***				0.078
Prospective	7	1.39 (1.13–1.72)	76.1%, < 0.001	
Retrospective	12	1.04 (0.89–1.23)	75.4%, < 0.001	
***1.4 Confounders adjusted***				0.601
Multivariate	15	1.15 (1.01–1.32)	87.4%, < 0.001	
Univariate	4	1.30 (0.93–1.81)	0.0%, 0.841	
***1.5 Quality***				0.684
< 7	8	1.23 (0.98–1.55)	44.3%, 0.083	
≥ 7	11	1.14 (0.97–1.33)	89.9%, < 0.001	
2. Age	7	0.85 (0.71–1.01)	59.0%, 0.023	

*p*-value* for Heterogeneity; *p*-value^#^ for meta regression

Seven studies reported data regarding relevance between different age stages and the incidence of PJI. The overall pooled RR and 95% CI (0.85, 0.71–1.01) were calculated using a random model and results exhibited heterogeneity (*I*^*2*^ = 59.0%, *P* = 0.023) in some degree (Fig. 3B). It indicates that more evidence is needed to confirm age as a risk factor of PJI.

### Effect of medical history characteristics on the incidence of PJI

**Fig. 4:**
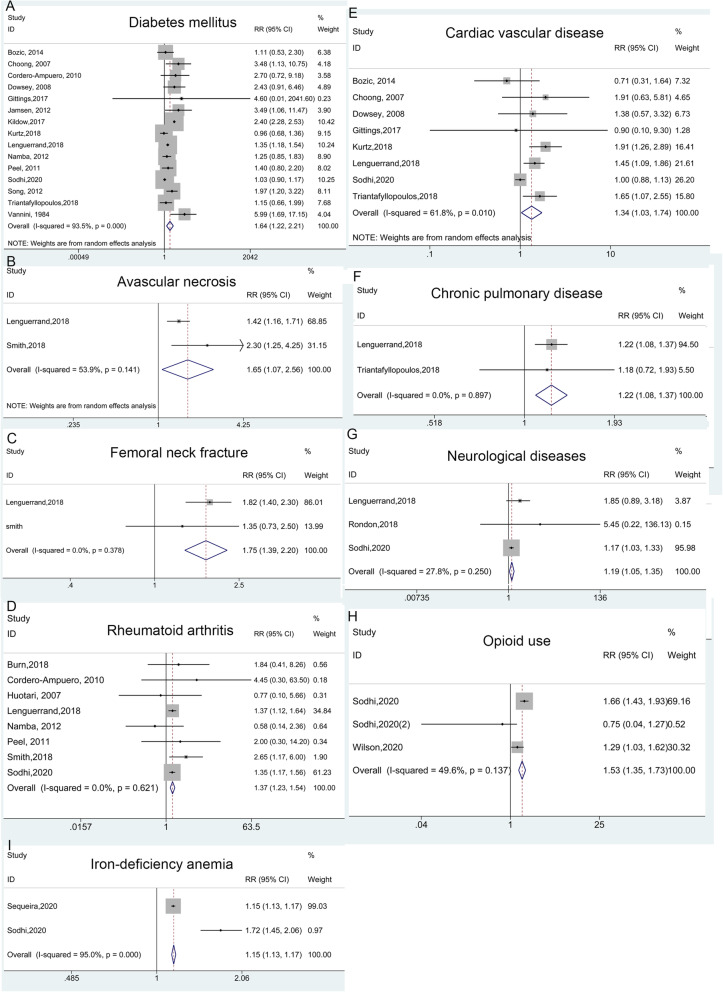
Forest plots of the meta-analysis of medical history characteristics, including diabetes mellitus (**A**), avascular necrosis (**B**), femoral neck fracture (**C**), rheumatoid arthritis (**D**), cardiac vascular disease (**E**), chronic pulmonary disease (**F**), neurological diseases (**G**), opioid use (**H**) and iron-deficiency anemia (**I**) as risk factors for PJI following THA

**Fig. 5:**
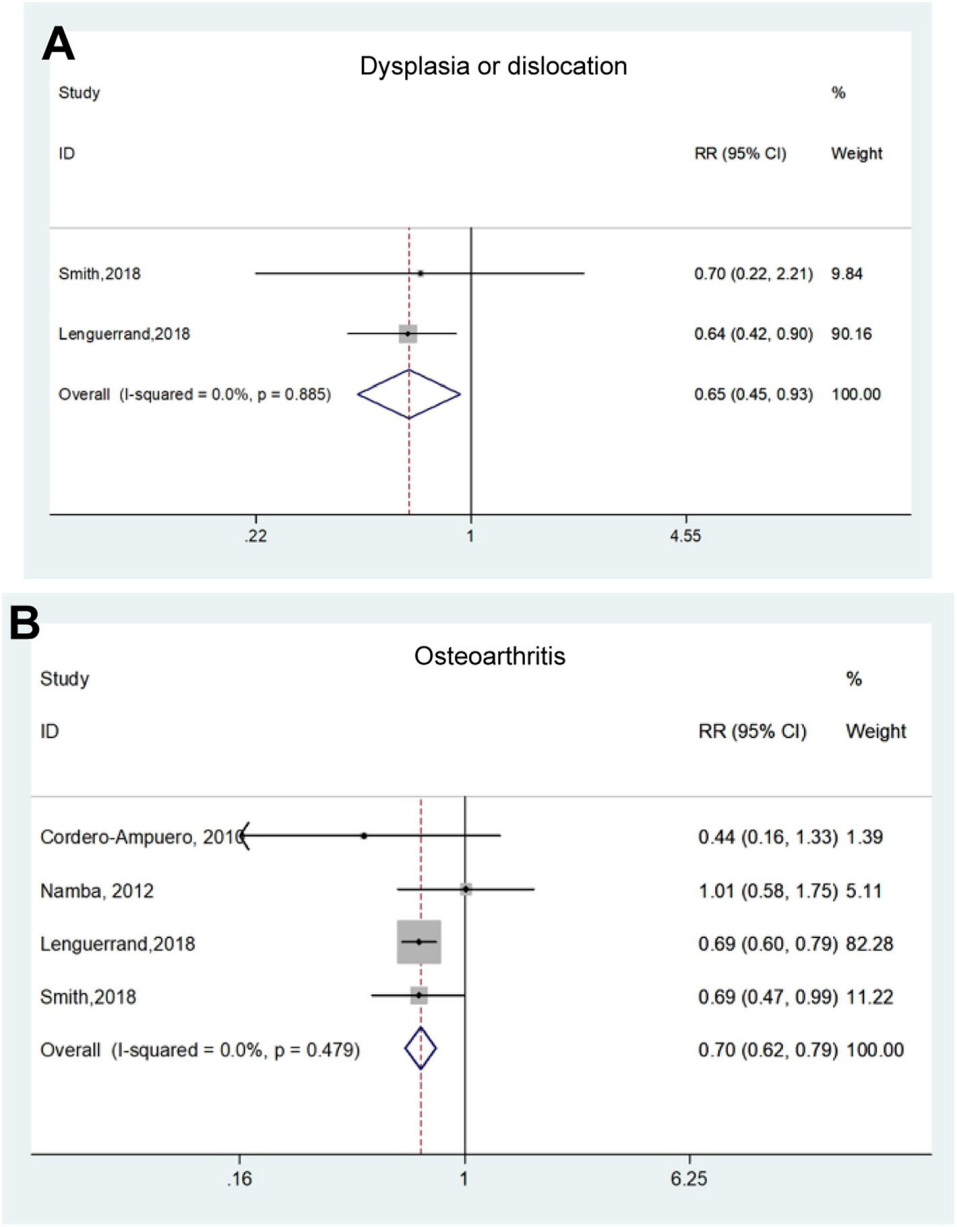
Forest plots of the meta-analysis of medical history characteristics, including dysplasia or dislocation (**A**), and osteoarthritis (**B**) as risk factors for PJI following THA

Seventeen medical history characteristics reported in studies were associated with PJI, and details were presented in Table 4. Sixteen studies exhibited the effect of diabetes mellitus (DM) on the incidence of PJI (Fig. 4A). The overall pooled RR and corresponding 95% CI (1.64, 1.22–2.21) was calculated using a random model and results showed obvious heterogeneity (*I*^*2*^ = 93.5%, *p* < 0.001). Similarly, subgroup analyses were performed to find potentially explicable variety of DM (Figure S2). Furthermore, meta-analysis results indicated that avascular necrosis (AVN) of femoral head (Fig. 4B), femoral neck fracture (Fig. 4C), rheumatoid arthritis (RA) (Fig. 4D), cardiac vascular disease (CVD) (Fig. 4E), chronic pulmonary disease (Fig. 4F), neurological diseases including dementia and Parkinson’s disease (Fig. 4G), opioid use (Fig. 4H) and iron-deficiency anemia (IDA) (Fig. 4I) were significant risk factors of the incidence of PJI after THA. While the comparisons about osteoarthritis (OA) vs. non-OA, and dysplasia or dislocation vs. non groups showed the opposite effects on PJI, implicating that OA and dysplasia/dislocation could be served as protective factors for PJI (Fig. 5). Additionally, there was not enough evidence to prove the previous joint surgery, renal disease, hypertension, cancer, steroid use history and liver disease associating with PJI (Figure S3). The details of the above pooled RRs, 95% CI and heterogeneity analysis were revealed in Table 4.

**Table 4:** Medical history characteristics for periprosthetic joint infection and the outcomes of meta-analysis

Comparisons	Number of studies	RR (95% CI)	Heterogeneity ***I***^**2**^, ***p***-value*	***p***-value^**#**^
1. DM vs. non-DM	15	1.64 (1.25–2.21)	93.5%, < 0.001	
***1.1 Location***				0.079
North America	7	1.28 (0.79–2.07)	96.5%, < 0.001	
Asia-Pacific	4	1.84 (1.34–2.53)	0.0%, 0.434	
Europe	4	2.59 (1.17–5.74)	68.8%, 0.022	
***1.2 Follow-up years***				0.253
≥ 3	5	1.24 (0.97–1.57)	38.9%, 0.162	
< 3	10	1.94 (1.28–2.93)	94.1%, < 0.001	
***1.3 Design***				0.942
Prospective	5	1.56 (1.09–2.25)	47.9%, 0.104	
Retrospective	10	1.64 (1.09–2.46)	94.7%, < 0.001	
***1.4 Confounders adjusted***				0.387
Multivariate	9	1. 45 (1.00–2.11)	96.1%, < 0.001	
Univariate	6	2.01 (1.28–3.16)	50.1%, 0.075	
2. AVN vs. non	2	1.65 (1.07–2.56)	53.9%, 0.141	
3. femoral neck fracture vs. non	2	1.75 (1.39–2.20)	0.0%, 0.378	
4. RA vs. non	8	1.37 (1.23–1.54)	0.0%, 0.621	
5. CVD vs. non	8	1.34 (1.03–1.74)	61.8%, 0.010	
6. Chronic pulmonary disease vs. non	2	1.22 (1.08–1.37)	0.0%, 0.897	
7. Neurological diseases vs. non	3	1.19 (1.05–1.35)	27.8%, 0.250	
8. Opioid use vs. non	3	1.53 (1.35–1.73)	49.6%, 0.137	
9. IDA vs. non	2	1.15 (1.13–1.17)	95%, < 0.001	
10. Dysplasia or dislocation vs. non	2	0.65 (0.45–0.93)	0.0%, 0.885	
11. OA vs. non	4	0.70 (0.62–0.79)	0.0%, 0.479	
12. Previous joint surgery vs. non	4	2.69 (0.67–10.72)	83.6%, < 0.001	
13. Renal disease vs. non	4	1.33 (0.50–3.53)	98.7%, < 0.001	
14. Hypertension vs. non	3	1.17 (0.94–1.46)	34.6%, 0.217	
15. Cancer vs. non	2	1.06 (0.87–1.29)	0.0%, 0.486	
16. Steroid use vs. non	6	1.80 (0.89–3.63)	9.2%, 0.357	
17. Liver disease vs. non	2	1.73 (0.86–3.51)	94.0%, < 0.001	

*p*-value* for Heterogeneity; *p*-value^#^ for meta regression; *AVN* Avascular necrosis, *CVD* Cardiac vascular disease, *DM* Diabetes, *IDA* Iron-deficiency anemia, *OA* Osteoarthritis, *RA* Rheumatoid arthritis, *IDA* Iron-deficiency anemia

### Sensitivity analysis

**Fig. 6:**
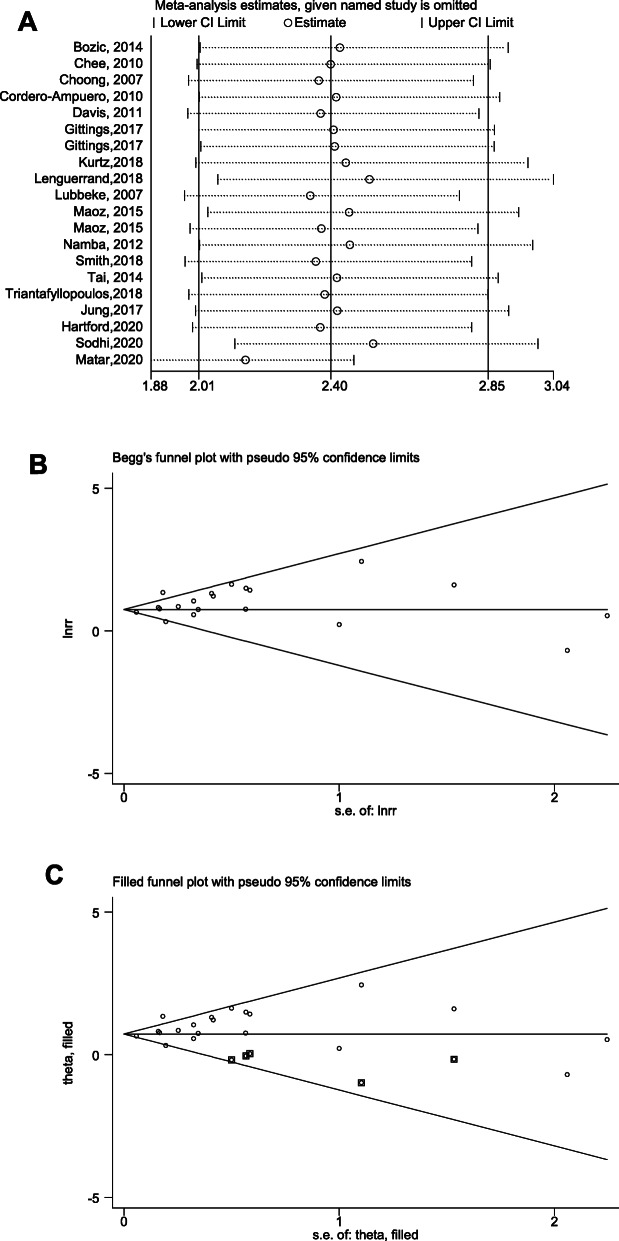
Sensitivity analysis (**A**), and Begg’s funnel plot (**B**) of the outcome: BMI. Funnel plot after modified with trimmed and filled method (**C**) of the outcome: BMI

Sensitivity analysis was performed to assess whether the pooled results were credible. In BMI comparison, sensitivity analysis illustrated that excluding the study by Matar, et al. [36] changed the original results and the modified pooled RR (95%CI) was 2.15 (1.88–2.46) after excluding this study (Fig. 6A). However, the significance of BMI in predicting PJI risk was not altered, indicating that high BMI was still positively correlated with increased PJI risk. Sensitivity analysis of gender factor was shown in Figure S1D.

### Publication Bias

Funnel plots, Egger’s and Begg’s tests were performed to evaluate the publication bias of the included studies, visually and statistically. BMI comparison had publication bias (*P* = 0.046 with Egger’s test). RR and 95% CI (2.228, 1.867–2.660) were modified with random trimming and filled method (Fig. 6B, C). In addition, Gender comparison did not have publication bias (*P* = 0.378 with Begg’s test) and DM comparison also did not have publication bias (*P* = 0.252 with Begg’s test). (Figure S1E, S2E).

## Discussion

Our meta-analysis focused on the patient-related factors associated with PJI, other than surgical- or hospital-related factors. The main finding of this meta-analysis was that the high BMI was the main risk factor for PJI. Additionally, DM and other comorbidities, such as AVN, RA, CVD, CPD, neurological disease, opioid use and IDA were also pivotal risk factors for PJI. In contrast, dysplasia or dislocation, OA were protective factors. Besides, female gender was protective after long follow-up duration (≥3 years). Moreover, age, smoking, alcohol abuse and other medical history such as previous joint surgery, renal disease, hypertension, cancer, steroid use and liver disease were not correlated with risk of PJI.

Obesity poses a major health challenge worldwide [1]. Consistent with previous finding [18, 61], our data showed that patients with a BMI greater than 40, 35, or 30 had a 2.99, 2.46, or 2.02–fold higher risk of PJI compared with those counterparts with less BMI, respectively. Obese patients are more prone to increased risk of PJI in the peri-operative setting, which may be attributed to prolonged operative and anesthetic time, higher colonization risk for *C. avidum* in the groin [11], longer hospital stay and high readmission rates within 30 days [9]. Besides, being obese is usually correlated with higher presence of other commodities including metabolic syndrome, wound dehiscence, and heart disease [1]. Notably, we found that the PJI risk increased exponentially along with BMI value. Similarly, Xu C et al. demonstrated that each one-unit increase in BMI was correlated with an 8% higher risk of PJI [4], thus the morbidly obese patients (BMI > 40 kg/m^2^) may have the highest likelihood of complications [13]. Another study also confirmed that all-cause revision rater after primary TJA doubled in patients with BMI ≥35 kg/m^2^, and even tripled in morbidly obese patients when compared with the controls [7, 62]. Accordingly, the increased risk of obesity should be weighed against the benefits of THA [7].

The relationship between DM and PJI has been well-established with a projected increase for years [18, 61, 63]. Our study showed that, comparing with non-DM patients, those undergoing THA with DM carried 1.69-fold greater risk of PJI (1.69, 1.26–2.28). Further stratified analyses, such as location, study design or analyses method, did not alter the unfavorable predictive value of DM on PJI risk. Epidemiological studies have identified that DM predisposes patients to PJI, probably due to increased infection rate of bacteria, impaired immune response, and postoperative hyperglycemia [64]. HbA1c is a widely used biomarker for diagnosis and monitoring of type 2 DM [17]. Recently, Cancienne JM et al. reviewed 7736 patients who underwent THA, and identified that HbA1c of 7.5 mg/dl could serve as a threshold for prediction of PJI after THA [10].

Both two studies reported the association between AVN or femoral neck fracture and PJI, and the pooled results indicated a 1.65 or 1.75-times increased risk of PJI than non-group, respectively. In contrast, four studies demonstrated the relationship between OA and PJI risk, and the calculated RR with corresponding CI was 0.70 (0.62–079), suggesting a protective role of OA in PJI. It is generally accepted that patients underwent THA for AVN are more likely to have readmission and surgical complications including bleeding transfusion [65]. Meanwhile, patients with femoral neck fracture undergoing THA also confer to higher rate of dislocation, infection and reoperation [66], which may be accountable for the increased risk of PJI [67]. OA refers to a common degenerative joint disease, and contribute to a majority cause for THA. However, compared with other commodities, patients with OA seem to have decreased occurrence of PJI after surgery. Another meta-analysis consisting of 37 studies reached a similar conclusion, showing that OA was protective factor in predicting PJI after THA/TKA [18]. Besides, Mayers W et al. searched the patients’ profiles with primary THA in US from 2001 to 2010, and found that patient underwent THA for OA have lesser medical complications and lower incidence for myocardial infarction than those with AVN [68].

RA and CVD have been reported as independent risk factors for PJI [3, 69]. Consistently, our results showed that PJI incidence were 1.37 or 1.34 times higher in patients reporting a history of RA or CVD, respectively. Patients with RA are more susceptible to PJI thought to be secondary to immune-suppressive therapies and poor nutritional conditions [18, 20]. Meanwhile, patient with CVD are recommended to receive aggressive anticoagulation therapy, such as aspirin or warfarin, may markedly increases risk of bleeding and wound hematoma after THA [1, 69], and thereby increase PJI rate [18].

Moreover, our study showed that patients with chronic pulmonary disease had 1.22–fold higher risk and with neurological disease had 1.19-fold higher risk (1.19, 1.05–1.35) of PJI when compared with non-group, which is in accordance with the published literature [3, 18, 69]. A meta-analysis conducted by Resende VAC et al. [18] showed that chronic lung disease significantly increased the risk for PJI after TJA. In addition to THA/TKA, another large multi-institutional retrospective study comprising 6977 patients also revealed a significant positive association between chronic lung disease and total ankle arthroplasties (TAA) [70]. More recently, a meta-analysis with eight studies enrolled further confirmed that increased risk of PJI following TAA for patients with lung disease [71].

Gender shows conflicting results among the selected studies. A number of studies have demonstrated that male patients are more vulnerable to PJI compared to women [18, 67]. Whereas other studies have refuted this, claiming women had an elevated risk of deep infection than men [48, 72]. Our results showed that gender was markedly correlated with PJI risk (1.17, 1.03–1.32) and female maybe a protective factor of PJI after THA. It is worth noting that male patients seem to coincide with higher incidence of unfavorable behavioral factors, which may result in increased risk of PJI.

The impact of age has been controversial among the enrolled studies. In general, older patients are more prone to postoperative infection caused by less competent immunity [5], malnutrition status [64] and complex medical comorbidities [8]. On the contrary, a multi-center analysis of 623,253 joint replacements claimed that younger age was closely correlated with elevated PJI [40], which may be attributed to active use cycles of implants, higher possibility of infection and revision surgery [64]. Besides, another meta-analysis with 2,470,827 patients also demonstrated older age was a protective factor in TJA [18]. Interestingly, we found that age had not direct influence on risk rate of PJI (0.85, 0.71–1.01), which was consistent with the results concluded by Kunutsor SK et al [73]. Recently, a single-center retrospective study comprising 23,966 patients also demonstrated that age alone was not a risk factor for PJI after adjusting for confounding variables [8], indicating that other covariates should be taken into account for assessing the relationship between age and PJI. Future studies are needed to further address these findings by adequately controlling the confounders.

Previous meta-analyses have demonstrated that smoking and alcohol abuse may result in increased risk PJI. Therefore, it is of great importance to encourage smoking and alcohol cessation during peri-operation period, and some reports suggested a 4–6 week cessation prior to surgery may be effective [1]. Specifically, excessive smoking was documented as to increase incidence of infection, mainly due to impaired wound-healing capacity, disrupted immune response, as well as nicotine or carbon monoxide-mediated vasoconstriction, soft-tissue hypo-perfusion, hypoxia, and thrombi formation [1]. While alcohol could affect the immune system and contribute to impaired phagocytic function [64]. In addition, alcohol abuse is a deleterious factor for developing cirrhosis, which may in turn increase risk of infection [64]. Intriguing, our pooled results showed that smoking and alcohol abuse were not correlated with PJI following THA. However, it should be noted that smoking may contribute to synergistic effect on elevated risk of PJI (3.54-fold) for patients with obesity [4, 74]. Accordingly, caution is still warranted when considering a THA in tobacco and alcohol users. More trials are still needed to extensively elucidate the underlying mechanism between smoking/ alcohol abuse and PJI.

It is imperative that both the surgeons and patients understand and identify the modifiable risk factors prior to THA so that they could make prudent decisions in the perioperative management, mitigate the risk of PJI, and therefore decrease the enormous financial and social burden of PJI [1, 9]. The findings in this meta-analysis, along with advanced diagnostic and treatment approaches, will allow the development of prevention strategies and increase the adherence to the clinical practice. Taken together, these results may offer help in developing guidelines on prevention of PJI after THA, and eventually establishing strategies to control it.

In addition to the factors discussed above, preoperative and intraoperative factors also play important roles. Nasal *Staphylococcus aureus* (*S. aureus*) decolonization by the strategies of antibiotic prophylaxis has been widely used to prevent PJI before THA. The influence of preoperative decolonization on PJI was discussed in a recent meta-analysis including 36,000 cases of primary THA and TKA. S. aureus screening and decolonization prior to primary THA were associated with a significantly decreased risk of surgical site infections and PJI [75]. Moreover, whether the fixation method influences the risk of PJI following THR is still controversial. A recent meta-analysis reported that all kinds of cemented fixations including plain, antibiotic and hybrid were each associated with an increased PJI risk when compared with uncemented fixations. In cemented fixations group, plain cemented fixations increased PJI risk compared to antibiotic-loaded cemented fixations [76, 77]. Furthermore, different prosthetic bearing surface materials may influence the risk of PJI after THA. A previous meta-analysis comprised 11 randomized controlled trials and six observational studies until September 2016 compared the rate of PJI between metal-on-polyethylene (MoP), ceramic-on-polyethylene (CoP), and ceramic-on-ceramic (CoC) bearings. It showed no significant difference between the three bearing combinations in terms of risk of PJI [78]. Although no bearing surfaces can deny the risk currently, subsequent articles containing large amounts of data have found that ceramic surfaces could reduce risk are convincing [40, 79, 80]. In addition, a prospective observational cohort study analyzing 623 253 primary THA investigated lateral surgical approach was associated with the increased risk of PJI from 3 months onwards patients compared to posterior surgical approach, due to the increased tissue damage and bleeding, probably [40].

However, it should be noted that some limitations remain to be addressed. First, most of the studies were retrospective, giving rise to a comparative low quality of evidence as per the NOS score. In addition, high proportion of retrospective studies would lead to inherent bias inevitably [81]. Second, number of included hips among the selected studies ranged from 33 to 1,158,742, the inclusion of these studies may lead to bias and confounding within our results. Third, some of the comparison like AVN of femoral head, femoral neck fracture, chronic pulmonary disease, IDA, dysplasia or dislocation of hip, cancer and liver disease included 2 studies only, respectively. It might limit the meaning of these pooled result. Fourth, the RRs were not adjusted to include more participants. Fifth, the follow-up time was heterogeneous among studies with a range from 0.25 to 10 years and did not differentiate time of onset of infection after primary arthroplasty. Sixth, the included studies were performed between 1983 to 2020. The THA operation technology in different period was significantly different, especially in two studies in 1983 and 1984.This maybe a reason for the bias of this meta-analysis. Seventh, the races vary among the selected studies, which may lead to discrepancies between various studies [64]. Lastly, another relevant issue and potential confounder of the results was the different diagnostic tools for PJI used in the original studies and the lack of a risk factor definition in the eligible studies, except for obesity was defined as BMI.

## Conclusion

Taken together, this meta-analysis identified significant risk factors for PJI associated with THA are high BMI, DM, AVN, femoral neck fracture, RA, CVD, chronic pulmonary disease, neurological disease, opioid use and IDA while protective factors include female gender, OA and dysplasia/ dislocation. We therefore suggest optimization of modifiable risk factors such as BMI for reducing the risk of PJI in clinical practice.

## Supplementary Information

**Additional file 1: Figure S1.** Subgroup analysis for gender with different location (A), Follow-up years (B), or confounders adjusted (C) were shown to identify the possible association between gender with PJI following THA. Sensitivity analysis (D), Begg’s funnel plot (E) of the outcome: gender. **Figure S2.** Subgroup analysis for diabetes mellitus (DM) with different location (A), Follow-up years (B), study design (C) or confounders adjusted (D) were shown to identify the possible association between DM with PJI after THA. Begg’s funnel plot (E) of the outcome: DM. **Figure S3.** Forest plots of the meta-analysis of medical history characteristics, including previous joint surgery (A), renal disease (B), hypertension (C), cancer (D), steroid use (E) and liver disease (F) as risk factors for PJI following THA.

## Data Availability

All data generated or analyzed during this meta-analysis were included in published article which were shown in Table 1. Comprehensive literature retrieval was performed from Pubmed, Web of Science, and the Cochrane Library. And the data from Pubmed, Web of Science, and the Cochrane Library are fully available without restriction.

## Abbreviations

AVN: Avascular necrosis
CIs: Corresponding intervals
CVD: Cardiovascular disease
DM: Diabetes mellitus
MOOSE: Meta-analysis of Observational Studies in Epidemiology
NOS: Newcastle-Ottawa Scale
OA: Osteoarthritis
ORs: Odds ratio
PJI: Periprosthetic joint infection
PRISMA: Preferred Reporting Items for Systematic reviews and Meta-Analysis
RA: Rheumatoid arthritis
RRs: Risk ratios
TAA: Total ankle arthroplasty
TKA: Total knee arthroplasty
THA: Total hip arthroplasty
THR: Total hip replacement
