# Supplementary material for: Patients’ risk factors for periprosthetic joint infection in primary total hip arthroplasty: a meta-analysis of 40 studies
Source: BMC Musculoskelet Disord. 2021 Sep 12;22:776. doi: 10.1186/s12891-021-04647-1 (PMC8436433; doi:10.1186/s12891-021-04647-1)
Supplement: Supplementary file 1 — Additional file 1: Figure S1. Subgroup analysis for gender with different location (A), Follow-up years (B), or confounders adjusted (C) were shown to identify the possible association between gender with PJI following THA. Sensitivity analysis (D), Begg’s funnel plot (E) of the outcome: gender. Figure S2. Subgroup analysis for diabetes mellitus (DM) with different location (A), Follow-up years (B), study design (C) or confounders adjusted (D) were shown to identify the possible association between DM with PJI after THA. Begg’s funnel plot (E) of the outcome: DM. Figure S3. Forest plots of the meta-analysis of medical history characteristics, including previous joint surgery (A), renal disease (B), hypertension (C), cancer (D), steroid use (E) and liver disease (F) as risk factors for PJI following THA. [file 12891_2021_4647_MOESM1_ESM.pdf]

Table S1. Methodological quality assessment of the included trials

Author	representativeness	comparability	ascertainment of outcome	Score
Bozic, 2014	3	2	2	7
Burn,2018	3	2	2	7
Chee, 2010	4	0	1	5
Choong, 2007	3	0	2	5
Cordero-Ampuero, 2010	3	0	2	5
Dale, 2011	3	2	2	7
Davis, 2011	4	2	2	8
de Boer, 2001	3	2	1	6
Dowsey, 2008	3	0	2	5
Gittings,2017	3	2	1	6
Gonzalez,2018	3	2	2	7
Hartford, 2020	3	1	2	6
Huotari, 2007	3	0	2	5
Jamsen, 2012	3	2	2	7
Jung	3	1	2	6
Kildow,2017	3	1	2	6
Kurtz,2018	3	2	2	7
Lenguerrand,2018	4	2	2	8
Lubbeke, 2007	4	2	1	7
Maoz, 2015	3	2	1	6
Marjo, 2007	4	2	1	7
Matar, 2020	3	1	2	6
McIntosh, 2006	3	2	1	6
Meermans, 2012	3	0	2	5
Muilwijk, 2006	3	2	2	7
Namba, 2012	3	2	2	7
Ong, 2009	4	2	1	7
Peel, 2011	3	0	2	5
Rondon,2018	3	1	2	6
Sequeira, 2020	3	2	2	7
Sequeira,S, 2020	4	1	1	6
Smith,2018	3	2	2	7
Sodhi, 2020	3	1	2	6
Sodhi,N 2020	3	2	2	7
Song, 2012	3	2	2	7
Surin, 1983	3	0	2	5
Tai, 2014	4	0	1	5
Triantafyllopoulos,2018	3	2	2	7
Vannini, 1984	3	0	2	5
Wilson 2020	3	1	1	5

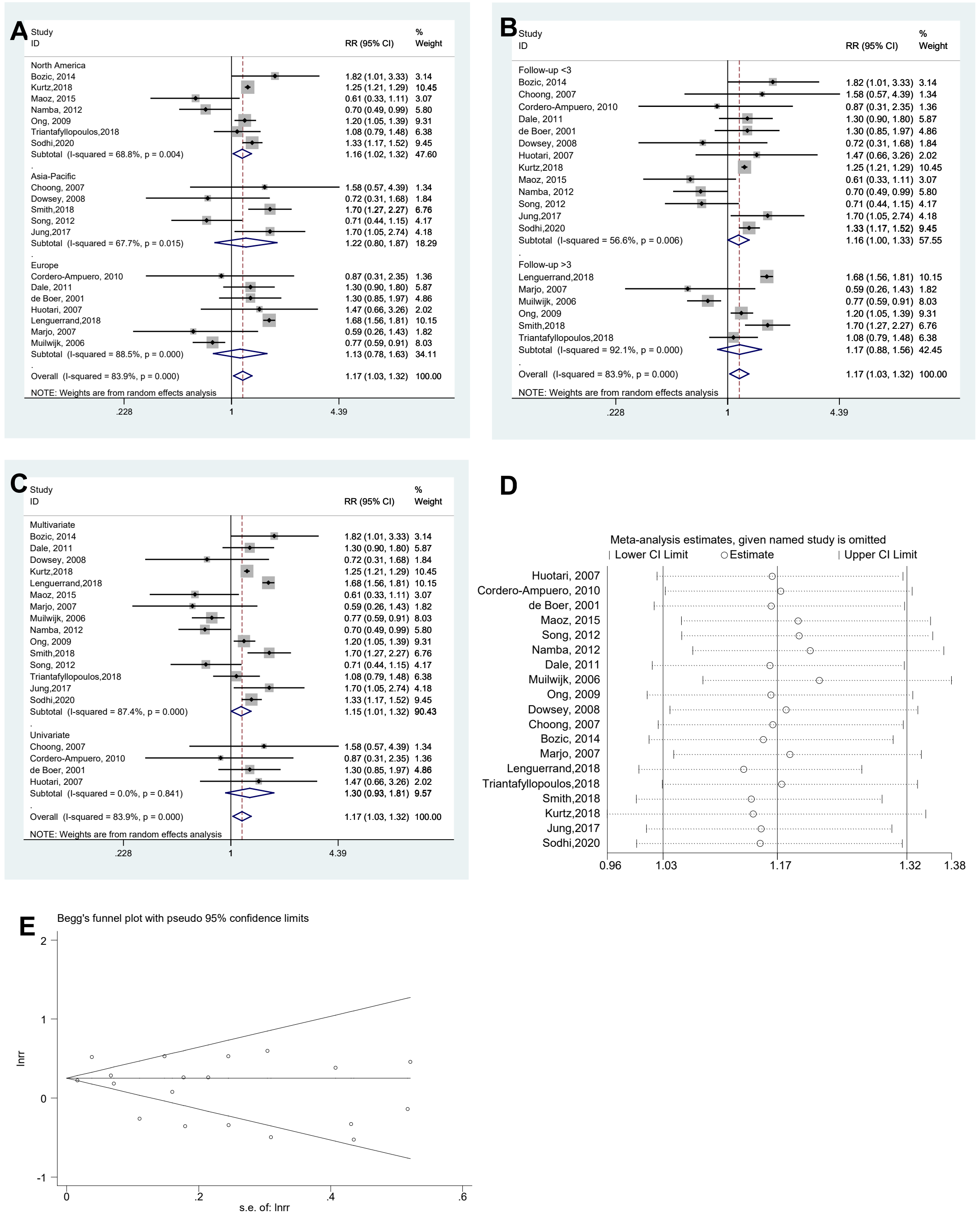
A
Study ID
RR (95% CI)
% Weight
North America
Bozic, 2014 1.82 (1.01, 3.33) 3.14
Kurtz,2018 1.25 (1.21, 1.29) 10.45
Maoz, 2015 0.61 (0.33, 1.11) 3.07
Namba, 2012 0.70 (0.49, 0.99) 5.80
Ong, 2009 1.20 (1.05, 1.39) 9.31
Triantafyllopoulos,2018 1.08 (0.79, 1.48) 6.38
Sodhi,2020 1.33 (1.17, 1.52) 9.45
Subtotal (I-squared = 68.8%, p = 0.004) 1.16 (1.02, 1.32) 47.60
Asia-Pacific
Choong, 2007 1.58 (0.57, 4.39) 1.34
Dowsey, 2008 0.72 (0.31, 1.68) 1.84
Smith,2018 1.70 (1.27, 2.27) 6.76
Song, 2012 0.71 (0.44, 1.15) 4.17
Jung,2017 1.70 (1.05, 2.74) 4.18
Subtotal (I-squared = 67.7%, p = 0.015) 1.22 (0.80, 1.87) 18.29
Europe
Cordero-Ampuero, 2010 0.87 (0.31, 2.35) 1.36
Dale, 2011 1.30 (0.90, 1.80) 5.87
de Boer, 2001 1.30 (0.85, 1.97) 4.86
Huotari, 2007 1.47 (0.66, 3.26) 2.02
Lenguerrand,2018 1.68 (1.56, 1.81) 10.15
Marjo, 2007 0.59 (0.26, 1.43) 1.82
Muilwijk, 2006 0.77 (0.59, 0.91) 8.03
Subtotal (I-squared = 88.5%, p = 0.000) 1.13 (0.78, 1.63) 34.11
Overall (I-squared = 83.9%, p = 0.000) 1.17 (1.03, 1.32) 100.00
NOTE: Weights are from random effects analysis
.228 1 4.39
B
Study ID
RR (95% CI)
% Weight
Follow-up <3
Bozic, 2014 1.82 (1.01, 3.33) 3.14
Choong, 2007 1.58 (0.57, 4.39) 1.34
Cordero-Ampuero, 2010 0.87 (0.31, 2.35) 1.36
Dale, 2011 1.30 (0.90, 1.80) 5.87
de Boer, 2001 1.30 (0.85, 1.97) 4.86
Dowsey, 2008 0.72 (0.31, 1.68) 1.84
Huotari, 2007 1.47 (0.66, 3.26) 2.02
Kurtz,2018 1.25 (1.21, 1.29) 10.45
Maoz, 2015 0.61 (0.33, 1.11) 3.07
Namba, 2012 0.70 (0.49, 0.99) 5.80
Song, 2012 0.71 (0.44, 1.15) 4.17
Jung,2017 1.70 (1.05, 2.74) 4.18
Sodhi,2020 1.33 (1.17, 1.52) 9.45
Subtotal (I-squared = 56.6%, p = 0.006) 1.16 (1.00, 1.33) 57.55
Follow-up >3
Lenguerrand,2018 1.68 (1.56, 1.81) 10.15
Marjo, 2007 0.59 (0.26, 1.43) 1.82
Muilwijk, 2006 0.77 (0.59, 0.91) 8.03
Ong, 2009 1.20 (1.05, 1.39) 9.31
Smith,2018 1.70 (1.27, 2.27) 6.76
Triantafyllopoulos,2018 1.08 (0.79, 1.48) 6.38
Subtotal (I-squared = 92.1%, p = 0.000) 1.17 (0.88, 1.56) 42.45
Overall (I-squared = 83.9%, p = 0.000) 1.17 (1.03, 1.32) 100.00
NOTE: Weights are from random effects analysis
.228 1 4.39
C
Study ID
RR (95% CI)
% Weight
Multivariate
Bozic, 2014 1.82 (1.01, 3.33) 3.14
Dale, 2011 1.30 (0.90, 1.80) 5.87
Dowsey, 2008 0.72 (0.31, 1.68) 1.84
Kurtz,2018 1.25 (1.21, 1.29) 10.45
Lenguerrand,2018 1.68 (1.56, 1.81) 10.15
Maoz, 2015 0.61 (0.33, 1.11) 3.07
Marjo, 2007 0.59 (0.26, 1.43) 1.82
Muilwijk, 2006 0.77 (0.59, 0.91) 8.03
Namba, 2012 0.70 (0.49, 0.99) 5.80
Ong, 2009 1.20 (1.05, 1.39) 9.31
Smith,2018 1.70 (1.27, 2.27) 6.76
Song, 2012 0.71 (0.44, 1.15) 4.17
Triantafyllopoulos,2018 1.08 (0.79, 1.48) 6.38
Jung,2017 1.70 (1.05, 2.74) 4.18
Sodhi,2020 1.33 (1.17, 1.52) 9.45
Subtotal (I-squared = 87.4%, p = 0.000) 1.15 (1.01, 1.32) 90.43
Univariate
Choong, 2007 1.58 (0.57, 4.39) 1.34
Cordero-Ampuero, 2010 0.87 (0.31, 2.35) 1.36
de Boer, 2001 1.30 (0.85, 1.97) 4.86
Huotari, 2007 1.47 (0.66, 3.26) 2.02
Subtotal (I-squared = 0.0%, p = 0.841) 1.30 (0.93, 1.81) 9.57
Overall (I-squared = 83.9%, p = 0.000) 1.17 (1.03, 1.32) 100.00
NOTE: Weights are from random effects analysis
.228 1 4.39
D
Meta-analysis estimates, given named study is omitted
Lower CI Limit
Estimate
Upper CI Limit
Huotari, 2007
Cordero-Ampuero, 2010
de Boer, 2001
Maoz, 2015
Song, 2012
Namba, 2012
Dale, 2011
Muilwijk, 2006
Ong, 2009
Dowsey, 2008
Choong, 2007
Bozic, 2014
Marjo, 2007
Lenguerrand,2018
Triantafyllopoulos,2018
Smith,2018
Kurtz,2018
Jung,2017
Sodhi,2020
0.96 1.03 1.17 1.32 1.38
E
Begg's funnel plot with pseudo 95% confidence limits
lnrr
2 1 0 -1
s.e. of: lnrr
0 .2 .4 .6

Figure S1.

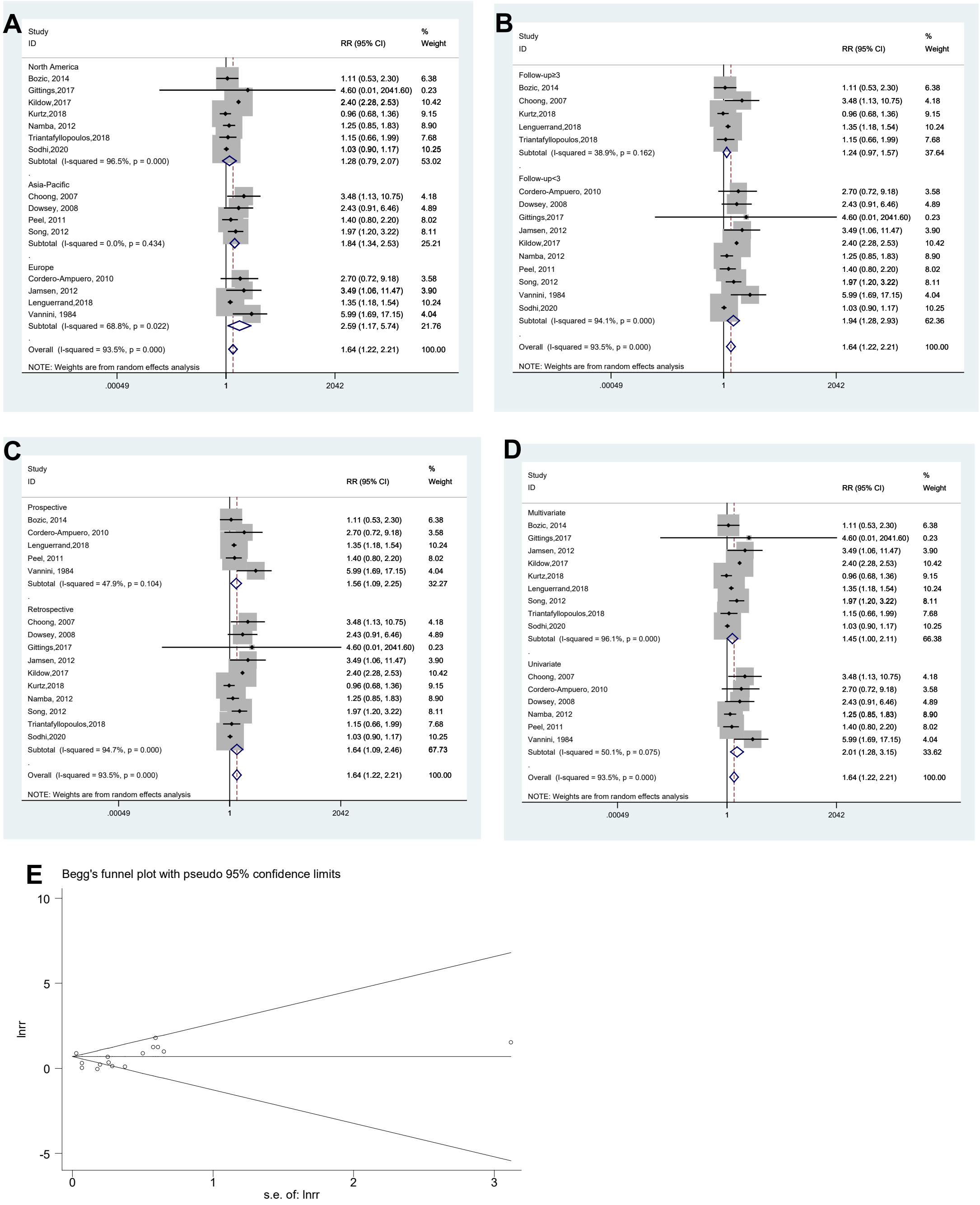

A
Study
ID
RR (95% CI)
% Weight
North America
Bozic, 2014 1.11 (0.53, 2.30) 6.38
Gittings,2017 4.60 (0.01, 2041.60) 0.23
Kildow,2017 2.40 (2.28, 2.53) 10.42
Kurtz,2018 0.96 (0.68, 1.36) 9.15
Namba, 2012 1.25 (0.85, 1.83) 8.90
Triantafyllopoulos,2018 1.15 (0.66, 1.99) 7.68
Sodhi,2020 1.03 (0.90, 1.17) 10.25
Subtotal (I-squared = 96.5%, p = 0.000) 1.28 (0.79, 2.07) 53.02
Asia-Pacific
Choong, 2007 3.48 (1.13, 10.75) 4.18
Dowsey, 2008 2.43 (0.91, 6.46) 4.89
Peel, 2011 1.40 (0.80, 2.20) 8.02
Song, 2012 1.97 (1.20, 3.22) 8.11
Subtotal (I-squared = 0.0%, p = 0.434) 1.84 (1.34, 2.53) 25.21
Europe
Cordero-Ampuero, 2010 2.70 (0.72, 9.18) 3.58
Jamsen, 2012 3.49 (1.06, 11.47) 3.90
Lenguerrand,2018 1.35 (1.18, 1.54) 10.24
Vannini, 1984 5.99 (1.69, 17.15) 4.04
Subtotal (I-squared = 68.8%, p = 0.022) 2.59 (1.17, 5.74) 21.76
Overall (I-squared = 93.5%, p = 0.000) 1.64 (1.22, 2.21) 100.00
NOTE: Weights are from random effects analysis
.00049 1 2042
B
Study
ID
RR (95% CI)
% Weight
Follow-up≥3
Bozic, 2014 1.11 (0.53, 2.30) 6.38
Choong, 2007 3.48 (1.13, 10.75) 4.18
Kurtz,2018 0.96 (0.68, 1.36) 9.15
Lenguerrand,2018 1.35 (1.18, 1.54) 10.24
Triantafyllopoulos,2018 1.15 (0.66, 1.99) 7.68
Subtotal (I-squared = 38.9%, p = 0.162) 1.24 (0.97, 1.57) 37.64
Follow-up<3
Cordero-Ampuero, 2010 2.70 (0.72, 9.18) 3.58
Dowsey, 2008 2.43 (0.91, 6.46) 4.89
Gittings,2017 4.60 (0.01, 2041.60) 0.23
Jamsen, 2012 3.49 (1.06, 11.47) 3.90
Kildow,2017 2.40 (2.28, 2.53) 10.42
Namba, 2012 1.25 (0.85, 1.83) 8.90
Peel, 2011 1.40 (0.80, 2.20) 8.02
Song, 2012 1.97 (1.20, 3.22) 8.11
Vannini, 1984 5.99 (1.69, 17.15) 4.04
Sodhi,2020 1.03 (0.90, 1.17) 10.25
Subtotal (I-squared = 94.1%, p = 0.000) 1.94 (1.28, 2.93) 62.36
Overall (I-squared = 93.5%, p = 0.000) 1.64 (1.22, 2.21) 100.00
NOTE: Weights are from random effects analysis
.00049 1 2042
C
Study
ID
RR (95% CI)
% Weight
Prospective
Bozic, 2014 1.11 (0.53, 2.30) 6.38
Cordero-Ampuero, 2010 2.70 (0.72, 9.18) 3.58
Lenguerrand,2018 1.35 (1.18, 1.54) 10.24
Peel, 2011 1.40 (0.80, 2.20) 8.02
Vannini, 1984 5.99 (1.69, 17.15) 4.04
Subtotal (I-squared = 47.9%, p = 0.104) 1.56 (1.09, 2.25) 32.27
Retrospective
Choong, 2007 3.48 (1.13, 10.75) 4.18
Dowsey, 2008 2.43 (0.91, 6.46) 4.89
Gittings,2017 4.60 (0.01, 2041.60) 0.23
Jamsen, 2012 3.49 (1.06, 11.47) 3.90
Kildow,2017 2.40 (2.28, 2.53) 10.42
Kurtz,2018 0.96 (0.68, 1.36) 9.15
Namba, 2012 1.25 (0.85, 1.83) 8.90
Song, 2012 1.97 (1.20, 3.22) 8.11
Triantafyllopoulos,2018 1.15 (0.66, 1.99) 7.68
Sodhi,2020 1.03 (0.90, 1.17) 10.25
Subtotal (I-squared = 94.7%, p = 0.000) 1.64 (1.09, 2.46) 67.73
Overall (I-squared = 93.5%, p = 0.000) 1.64 (1.22, 2.21) 100.00
NOTE: Weights are from random effects analysis
.00049 1 2042
D
Study
ID
RR (95% CI)
% Weight
Multivariate
Bozic, 2014 1.11 (0.53, 2.30) 6.38
Gittings,2017 4.60 (0.01, 2041.60) 0.23
Jamsen, 2012 3.49 (1.06, 11.47) 3.90
Kildow,2017 2.40 (2.28, 2.53) 10.42
Kurtz,2018 0.96 (0.68, 1.36) 9.15
Lenguerrand,2018 1.35 (1.18, 1.54) 10.24
Song, 2012 1.97 (1.20, 3.22) 8.11
Triantafyllopoulos,2018 1.15 (0.66, 1.99) 7.68
Sodhi,2020 1.03 (0.90, 1.17) 10.25
Subtotal (I-squared = 96.1%, p = 0.000) 1.45 (1.00, 2.11) 66.38
Univariate
Choong, 2007 3.48 (1.13, 10.75) 4.18
Cordero-Ampuero, 2010 2.70 (0.72, 9.18) 3.58
Dowsey, 2008 2.43 (0.91, 6.46) 4.89
Namba, 2012 1.25 (0.85, 1.83) 8.90
Peel, 2011 1.40 (0.80, 2.20) 8.02
Vannini, 1984 5.99 (1.69, 17.15) 4.04
Subtotal (I-squared = 50.1%, p = 0.075) 2.01 (1.28, 3.15) 33.62
Overall (I-squared = 93.5%, p = 0.000) 1.64 (1.22, 2.21) 100.00
NOTE: Weights are from random effects analysis
.00049 1 2042
E
Begg's funnel plot with pseudo 95% confidence limits
10
5
0
-5
lnrr
0
1
2
3
s.e. of: lnrr

Figure S2

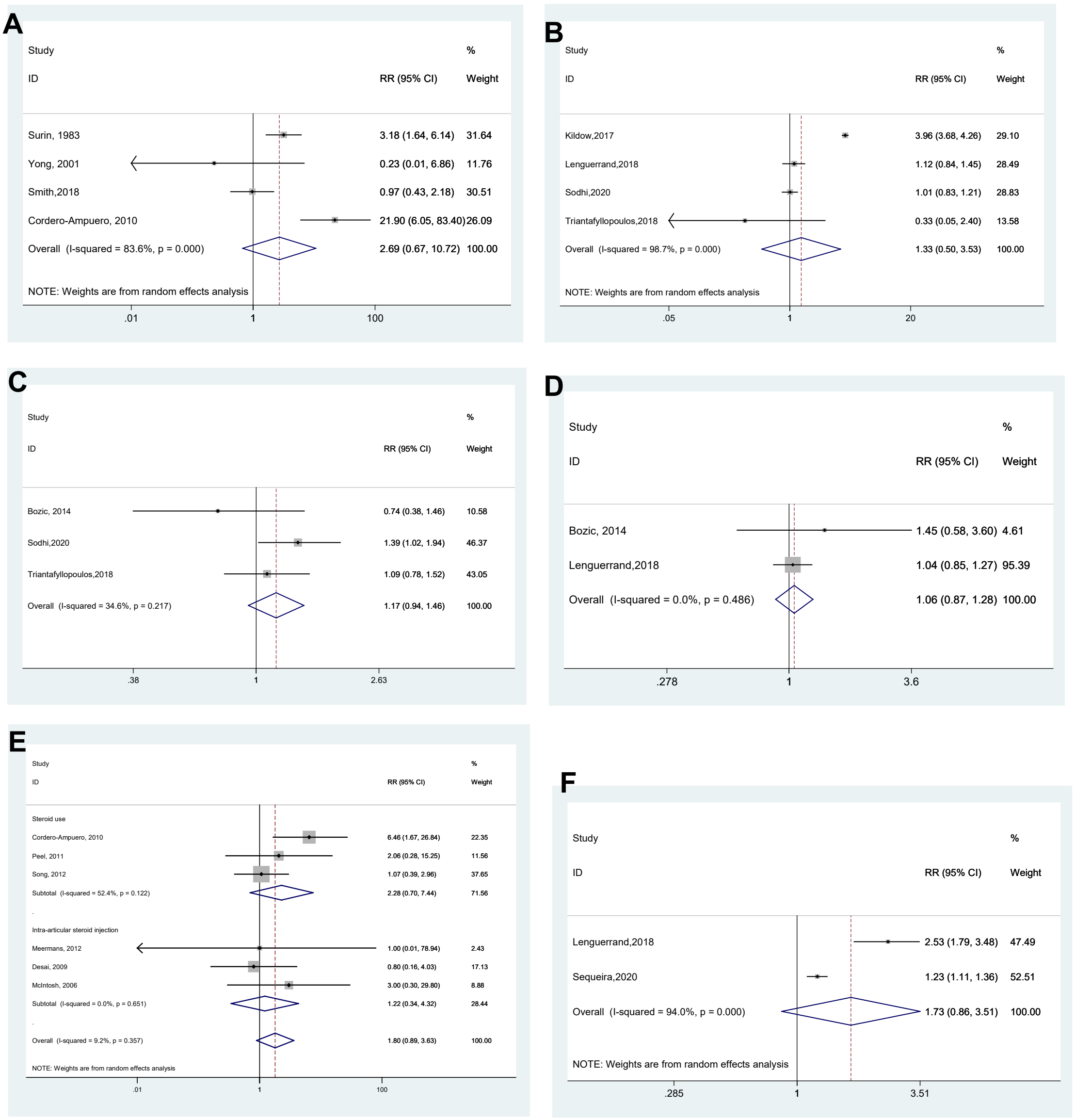
A
Study
ID
RR (95% CI)
%
Weight
Surin, 1983
3.18 (1.64, 6.14)
31.64
Yong, 2001
0.23 (0.01, 6.86)
11.76
Smith,2018
0.97 (0.43, 2.18)
30.51
Cordero-Ampuero, 2010
21.90 (6.05, 83.40)
26.09
Overall (I-squared = 83.6%, p = 0.000)
2.69 (0.67, 10.72)
100.00
NOTE: Weights are from random effects analysis
.01
1
100
B
Study
ID
RR (95% CI)
%
Weight
Kildow,2017
3.96 (3.68, 4.26)
29.10
Lenguerrand,2018
1.12 (0.84, 1.45)
28.49
Sodhi,2020
1.01 (0.83, 1.21)
28.83
Triantafyllopoulos,2018
0.33 (0.05, 2.40)
13.58
Overall (I-squared = 98.7%, p = 0.000)
1.33 (0.50, 3.53)
100.00
NOTE: Weights are from random effects analysis
.05
1
20
C
Study
ID
RR (95% CI)
%
Weight
Bozic, 2014
0.74 (0.38, 1.46)
10.58
Sodhi,2020
1.39 (1.02, 1.94)
46.37
Triantafyllopoulos,2018
1.09 (0.78, 1.52)
43.05
Overall (I-squared = 34.6%, p = 0.217)
1.17 (0.94, 1.46)
100.00
.38
1
2.63
D
Study
ID
RR (95% CI)
%
Weight
Bozic, 2014
1.45 (0.58, 3.60)
4.61
Lenguerrand,2018
1.04 (0.85, 1.27)
95.39
Overall (I-squared = 0.0%, p = 0.486)
1.06 (0.87, 1.28)
100.00
.278
1
3.6
E
Study
ID
RR (95% CI)
%
Weight
Steroid use
Cordero-Ampuero, 2010
6.46 (1.67, 26.84)
22.35
Peel, 2011
2.06 (0.28, 15.25)
11.56
Song, 2012
1.07 (0.39, 2.96)
37.65
Subtotal (I-squared = 52.4%, p = 0.122)
2.28 (0.70, 7.44)
71.56
Intra-articular steroid injection
Meermans, 2012
1.00 (0.01, 78.94)
2.43
Desai, 2009
0.80 (0.16, 4.03)
17.13
McIntosh, 2006
3.00 (0.30, 29.80)
8.88
Subtotal (I-squared = 0.0%, p = 0.651)
1.22 (0.34, 4.32)
28.44
Overall (I-squared = 9.2%, p = 0.357)
1.80 (0.89, 3.63)
100.00
NOTE: Weights are from random effects analysis
.01
1
100
F
Study
ID
RR (95% CI)
%
Weight
Lenguerrand,2018
2.53 (1.79, 3.48)
47.49
Sequeira,2020
1.23 (1.11, 1.36)
52.51
Overall (I-squared = 94.0%, p = 0.000)
1.73 (0.86, 3.51)
100.00
NOTE: Weights are from random effects analysis
.285
1
3.51

Figure S3
